# A new mono-functionalized organoimido hexa­molybdate derivative: bis­(tetra-*n*-butyl­ammonium) (5-chloro-2-methyl­phenyl­imido)-μ_6_-oxido-dodeca-μ_2_-oxido-penta­oxidohexa­molybdate(VI)

**DOI:** 10.1107/S1600536811036063

**Published:** 2011-09-20

**Authors:** Qiang Li, Zichen Xiao, Liye Chen, Jin Zhang

**Affiliations:** a Department of Chemistry, College of Science of Beijing Forestry University, Beijing 100083, People’s Republic of China; b Department of Chemistry, Tsinghua University, Beijing 100084, People’s Republic of China

## Abstract

The title complex, [(C_4_H_9_)_4_N]_2_[Mo_6_(C_7_H_6_ClN)O_18_], was prepared by the reaction of (Bu_4_N)_4_[*α*-Mo_8_O_26_] and 2-methyl-5-chloro­aniline hydro­chloride with *N*,*N*′-dicyclo­hexyl­carbodiimide as dehydrating agent in dry acetonitrile solution. The aryl­imido ligand is linked to an Mo atom of the Lindqvist-type hexamolybdate anion by an Mo N triple bond, with a bond length of 1.732 (4) Å and an Mo N—C bond angle of 169.1 (4)°, typical for monodentate imido groups in such hybrid complexes. Due to the inter­action between one H atom in the aryl group and an O atom of a symmetry-related hexa­molybdate cluster, the anions form centrosymmetric dimers in the crystal structure. Weak C—H⋯O contacts are observed between the cations and anions. Unresolved disorder in some of the butyl chains of the ammonium cation is noted.

## Related literature

For general background to polyoxidometalates, see: Hill & White (1998 ▶); Gili *et al.* (2000 ▶). For details of the synthesis, see: Wu *et al.* (2004 ▶). For related structures, see: Li *et al.* (2008 ▶). For organoimido polyoxidometalate derivatives, see: Du *et al.* (1992 ▶); Proust *et al.* (1994 ▶); Clegg *et al.* (1995 ▶). For Mo N triple bonds, see: Wigley (1994 ▶); Li *et al.* (2004 ▶).
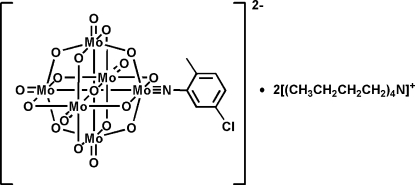


## Experimental

### 

#### Crystal data

(C_16_H_36_N)_2_[Mo_6_(C_7_H_6_ClN)O_18_]
*M* *_r_* = 1488.13
Monoclinic, 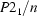

*a* = 12.9184 (9) Å
*b* = 20.7309 (16) Å
*c* = 20.6731 (15) Å
β = 94.077 (1)°
*V* = 5522.5 (7) Å^3^
*Z* = 4
Mo *K*α radiation
μ = 1.44 mm^−1^
*T* = 292 K
0.30 × 0.20 × 0.10 mm

#### Data collection

Bruke SMART APEX CCD area-detector diffractometer
Absorption correction: multi-scan (*SADABS*; Bruker, 2001 ▶) *T*_min_ = 0.673, *T*_max_ = 0.870
33748 measured reflections
10841 independent reflections
6533 reflections with *I* > 2σ(*I*)
*R*_int_ = 0.071

#### Refinement

*R*[*F* ^2^ > 2σ(*F* ^2^)] = 0.049
*wR*(*F* ^2^) = 0.118
*S* = 0.93
10841 reflections
604 parameters
3 restraints
H-atom parameters constrained
Δρ_max_ = 0.89 e Å^−3^
Δρ_min_ = −0.51 e Å^−3^

### 

Data collection: *SMART* (Bruker, 2001 ▶); cell refinement: *SAINT* (Bruker, 2001 ▶); data reduction: *SAINT*; program(s) used to solve structure: *SHELXS97* (Sheldrick, 2008 ▶); program(s) used to refine structure: *SHELXL97* (Sheldrick, 2008 ▶); molecular graphics: *SHELXTL* (Sheldrick, 2008 ▶); software used to prepare material for publication: *SHELXTL*.

## Supplementary Material

Crystal structure: contains datablock(s) global, I. DOI: 10.1107/S1600536811036063/bh2377sup1.cif

Structure factors: contains datablock(s) I. DOI: 10.1107/S1600536811036063/bh2377Isup2.hkl

Additional supplementary materials:  crystallographic information; 3D view; checkCIF report

## Figures and Tables

**Table 1:** Hydrogen-bond geometry (Å, °)

*D*—H⋯*A*	*D*—H	H⋯*A*	*D*⋯*A*	*D*—H⋯*A*
C5—H5⋯O3^i^	0.93	2.60	3.447 (7)	153
C8—H8*A*⋯O15^ii^	0.97	2.44	3.396 (6)	169
C16—H16*A*⋯O16^ii^	0.97	2.55	3.410 (7)	147
C12—H12*A*⋯O9	0.97	2.34	3.248 (7)	155

Symmetry codes: (i) 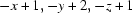; (ii) 
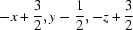
.

**Fig. 1.**
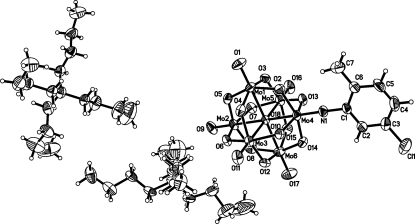
ORTEP showing the cluster anion and cations of the title component. Thermal ellipsoids are drawn at the 50% probability level.

**Fig. 2.**
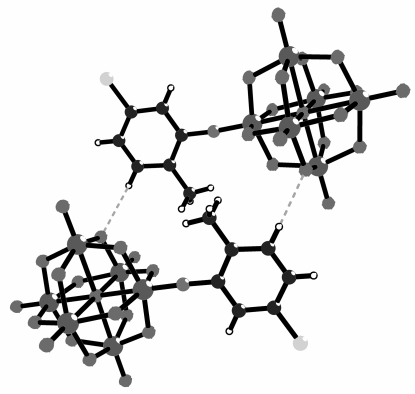
A view of the dimer structure of cluster anions of the title compound.

## supplementary crystallographic information

### Comment

The new organoimido derivatives of polyoxometalates have attracted tremendous attention due to the diversity of their structures. They are important building blocks for the construction of interesting POM-organic hybrids and have potential applications in nano-materials, magnetism, catalysis, photochemistry and medicine science (Hill & White, 1998; Gili *et al.*, 2000). Based on the pioneering works of E. A. Maatta (Du *et al.*, 1992), R. J. Errington (Clegg *et al.*, 1995) and A. Proust (Proust *et al.*, 1994), a great number of organoimido derivatives of polyoxometalates have been obtained, including alkyl and aryl derivatives of polyoxometalates. Particularly, chloro-functionalized organoimido derivatives of polyoxometalates are very useful in the construction of various POM-organic hybrids, which are more easily accessible and much cheaper than the corresponding iodide and bromide derivatives. Chloro derivatives are reactive functional groups, which are very useful in some organic syntheses. Recently, we have obtained three chloro-functionalized arylimido derivatives of hexamolybdate, (Bu_4_N)_2_[Mo_6_O_18_(≡N*R*)] (*R* = *p*-ClC_6_H_4_, *m*-ClC_6_H_4_, and *o*-ClC_6_H_4_) in moderate yields (Li *et al.*, 2008). However, they are not stable enough to undergo reactions in the subsequent synthesis process due to the electron-withdrawing nature of the chloro group and lack of protection of the Mo≡N bond, which is easily hydrolyzed in acid or alkaline media. In order to obtain more stable building blocks to construct novel POM-based organic-inorganic hybrids, we probed 2-CH_3_-5-ClC_6_H_3_NH_2_ as a ligand to modify the hexamolybdate ion, in which a methyl group *ortho* to the imido N atom on the benzene ring not only increases the stability of the resulting imido derivative, but also improves the yield of the synthesis.

X-ray diffraction analysis reveals that the title compound crystallizes in the monoclinic space group *P*2_1_/*n*. The asymmetric unit contains one crystallographically independent [Mo_6_O_18_N(2—CH_3_-5-ClC_6_H_3_)]^2-^ anion and two (C_4_H_9_)_4_N^+^ cations (Fig. 1). In the [Mo_6_O_18_N(2—CH_3_-5-ClC_6_H_3_)]^2-^ anion, an arylimido ligand is bound to one terminal position at the hexamolybdate cluster in a monodentate fashion. The short Mo—N bond distance, 1.732 (4) Å, and approximately linear C—N—Mo angle, 169.1 (4)°, are typical of organoimido groups bonded at an octahedral *d*^0^ metal center, and are consistent with a substantial degree of Mo≡N triple bond character (Wigley, 1994). Compared to the reported chloro-functionalized arylimido derivatives of hexamolybdate (Li *et al.*, 2008), the length of the Mo—N triple bond becomes larger (> 1.70 Å), and the C—N—Mo bond angle is closer to 180°, as found in other imido derivatives of Lindqvist polyoxometalates. The bond lengths of the five terminal oxo ligands do not vary significantly in comparison with the parent hexamolybdate and other derivatives. The central *µ*_6_-O atom O18 is displaced towards Mo4, as a consequence of the substitution of Mo4 by the arylimido ligand. Similar contraction has also been observed in the structures of other organoimido derivatives of Lindqvist polyoxometalates (Li *et al.*, 2004). Considerable variations are seen in the bond lengths involving the doubly bridging O atoms, which is again consistent with other imido derivatives of Lindqvist polyoxometalates (Li *et al.*, 2004).

An interesting feature is the solid phase dimerization of the cluster anions of the title compound, through C—H···O hydrogen bonds between a H atom on the aromatic ring and a bridging O atom in a symmetry-related anion (Fig. 2). Such a structural feature has also been observed before in the reported phenylimido derivatives of hexamolybdate (Wu *et al.*, 2004).

### Experimental

A mixture of (Bu_4_N)_4_[α-Mo_8_O_26_] (1.0 mmol), DCC (2.1 mmol), and 2-methyl-5-chloroaniline hydrochloride (1.34 mmol) was refluxed in anhydrous acetonitrile (10 ml) for about 12 h. After being cooled down to room temperature, the resulting dark-red solution was filtrated to remove the white precipitates. While most of acetonitrile evaporated, the product was collected from the filtrate as a red crystalline solid, and was washed successively with ethanol and ether several times, and then was recrystallized twice from a mixture of acetone and ethanol (1:1), to get red crystals (yield: 85 to 95%). Single crystals used for X-ray diffraction were obtained by diffusion of ether into a solution of the title compound in acetone.

### Refinement

All H atoms were positioned geometrically and allowed to ride on their parent atoms, with C–-H = 0.93–0.97 Å and *U*_iso_(H) = 1.2 or 1.5 *U*_eq_(parent atom). Bond lengths C14—C15, C18—C19 and C34—C35 were restrained to suitable target values.

### Crystal data

**Table:**

(C_16_H_36_N)_2_[Mo_6_(C_7_H_6_ClN)O_18_]	*F*(000) = 2984
*M**_r_* = 1488.13	*D*_x_ = 1.790 Mg m^−^^3^
Monoclinic, *P*2_1_/*n*	Mo *K*α radiation, λ = 0.71073 Å
Hall symbol: -P 2ybc	Cell parameters from 6101 reflections
*a* = 12.9184 (9) Å	θ = 2.2–24.7°
*b* = 20.7309 (16) Å	µ = 1.44 mm^−^^1^
*c* = 20.6731 (15) Å	*T* = 292 K
β = 94.077 (1)°	Prism, red
*V* = 5522.5 (7) Å^3^	0.30 × 0.20 × 0.10 mm
*Z* = 4	

### Data collection

**Table:**

Bruke SMART APEX CCD area-detector diffractometer	10841 independent reflections
Radiation source: fine-focus sealed tube	6533 reflections with *I* > 2σ(*I*)
graphite	*R*_int_ = 0.071
φ and ω scans	θ_max_ = 26.0°, θ_min_ = 1.4°
Absorption correction: multi-scan (*SADABS*; Bruker, 2001)	*h* = −15→10
*T*_min_ = 0.673, *T*_max_ = 0.870	*k* = −25→25
33748 measured reflections	*l* = −23→25

### Refinement

**Table:**

Refinement on *F*^2^	Primary atom site location: structure-invariant direct methods
Least-squares matrix: full	Secondary atom site location: difference Fourier map
*R*[*F*^2^ > 2σ(*F*^2^)] = 0.049	Hydrogen site location: inferred from neighbouring sites
*wR*(*F*^2^) = 0.118	H-atom parameters constrained
*S* = 0.93	*w* = 1/[σ^2^(*F*_o_^2^) + (0.0498*P*)^2^] where *P* = (*F*_o_^2^ + 2*F*_c_^2^)/3
10841 reflections	(Δ/σ)_max_ = 0.001
604 parameters	Δρ_max_ = 0.89 e Å^−^^3^
3 restraints	Δρ_min_ = −0.51 e Å^−^^3^
0 constraints	

### Fractional atomic coordinates and isotropic or equivalent isotropic displacement parameters (Å^2^)

**Table:**

	*x*	*y*	*z*	*U*_iso_*/*U*_eq_	
Mo1	0.58494 (4)	0.75656 (2)	0.49709 (2)	0.05960 (15)	
Mo2	0.70125 (3)	0.66987 (2)	0.61349 (3)	0.05930 (15)	
Mo3	0.44618 (3)	0.67834 (2)	0.59982 (2)	0.05393 (15)	
Mo4	0.46474 (3)	0.83417 (2)	0.60484 (2)	0.05293 (14)	
Mo5	0.71580 (4)	0.82789 (2)	0.61932 (3)	0.06038 (16)	
Mo6	0.57715 (4)	0.74969 (2)	0.72164 (2)	0.06564 (16)	
O1	0.5847 (3)	0.7566 (2)	0.41610 (19)	0.0978 (16)	
O2	0.4897 (3)	0.82359 (16)	0.51522 (16)	0.0623 (10)	
O3	0.6942 (3)	0.81455 (16)	0.52686 (17)	0.0662 (10)	
O4	0.4731 (2)	0.69632 (17)	0.51166 (16)	0.0605 (9)	
O5	0.6769 (2)	0.68730 (16)	0.52241 (17)	0.0633 (10)	
O6	0.5672 (2)	0.62595 (15)	0.60452 (17)	0.0619 (10)	
O7	0.7853 (3)	0.74421 (15)	0.61916 (19)	0.0644 (10)	
O8	0.6735 (3)	0.68276 (18)	0.70184 (17)	0.0703 (11)	
O9	0.7870 (3)	0.60842 (17)	0.6162 (2)	0.0909 (13)	
O10	0.3742 (2)	0.76027 (14)	0.59977 (16)	0.0529 (9)	
O11	0.3477 (3)	0.62517 (18)	0.5932 (2)	0.0842 (13)	
O12	0.4691 (3)	0.69127 (16)	0.69218 (16)	0.0632 (10)	
O13	0.5988 (3)	0.88071 (16)	0.61391 (17)	0.0679 (11)	
O14	0.4863 (3)	0.81790 (16)	0.69764 (16)	0.0640 (10)	
O15	0.6914 (3)	0.80787 (19)	0.70742 (18)	0.0742 (11)	
O16	0.8172 (3)	0.87935 (19)	0.6272 (2)	0.0953 (14)	
O17	0.5776 (4)	0.7478 (2)	0.8028 (2)	0.1080 (17)	
O18	0.5796 (2)	0.75430 (12)	0.60907 (14)	0.0420 (8)	
N1	0.3821 (3)	0.8999 (2)	0.6049 (2)	0.0644 (12)	
Cl1	0.21230 (17)	1.05088 (11)	0.76058 (11)	0.1326 (8)	
C1	0.3300 (4)	0.9579 (2)	0.6132 (3)	0.0616 (15)	
C2	0.2984 (4)	0.9730 (3)	0.6751 (3)	0.0708 (16)	
H2	0.3109	0.9445	0.7095	0.085*	
C3	0.2490 (5)	1.0303 (3)	0.6837 (3)	0.0815 (19)	
C4	0.2300 (6)	1.0724 (3)	0.6341 (4)	0.106 (2)	
H4	0.1955	1.1110	0.6406	0.127*	
C5	0.2623 (5)	1.0574 (3)	0.5741 (4)	0.102 (2)	
H5	0.2511	1.0874	0.5409	0.123*	
C6	0.3115 (5)	0.9993 (3)	0.5606 (3)	0.0748 (17)	
C7	0.3456 (5)	0.9835 (3)	0.4947 (3)	0.113 (2)	
H7A	0.2858	0.9782	0.4649	0.170*	
H7B	0.3852	0.9443	0.4969	0.170*	
H7C	0.3877	1.0180	0.4801	0.170*	
C8	0.6540 (4)	0.3553 (3)	0.6615 (3)	0.0732 (18)	
H8A	0.6898	0.3415	0.7019	0.088*	
H8B	0.5846	0.3374	0.6600	0.088*	
C9	0.7086 (5)	0.3264 (3)	0.6072 (3)	0.0764 (18)	
H9A	0.7810	0.3393	0.6112	0.092*	
H9B	0.6781	0.3429	0.5662	0.092*	
C10	0.7019 (5)	0.2527 (3)	0.6069 (4)	0.094 (2)	
H10A	0.7260	0.2369	0.6495	0.112*	
H10B	0.6297	0.2403	0.5992	0.112*	
C11	0.7625 (6)	0.2206 (3)	0.5579 (4)	0.128 (3)	
H11A	0.7404	0.2364	0.5155	0.191*	
H11B	0.7515	0.1748	0.5595	0.191*	
H11C	0.8349	0.2297	0.5670	0.191*	
C12	0.7496 (5)	0.4580 (3)	0.6520 (3)	0.0839 (19)	
H12A	0.7423	0.5045	0.6492	0.101*	
H12B	0.7712	0.4429	0.6106	0.101*	
C13	0.8344 (6)	0.4421 (4)	0.7044 (4)	0.115 (3)	
H13A	0.8480	0.3961	0.7070	0.138*	
H13B	0.8177	0.4580	0.7466	0.138*	
C14	0.9357 (9)	0.4833 (6)	0.6754 (6)	0.216 (6)	
H14A	0.9287	0.4857	0.6284	0.259*	
H14B	0.9400	0.5267	0.6929	0.259*	
C15	1.0190 (10)	0.4488 (7)	0.6957 (7)	0.285 (8)	
H15A	1.0189	0.4418	0.7416	0.427*	
H15B	1.0807	0.4718	0.6863	0.427*	
H15C	1.0174	0.4080	0.6737	0.427*	
C16	0.6076 (5)	0.4481 (3)	0.7271 (3)	0.0838 (19)	
H16A	0.6562	0.4315	0.7610	0.101*	
H16B	0.5413	0.4274	0.7320	0.101*	
C17	0.5952 (6)	0.5190 (4)	0.7374 (3)	0.113 (3)	
H17A	0.5423	0.5362	0.7065	0.135*	
H17B	0.6600	0.5411	0.7314	0.135*	
C18	0.5639 (8)	0.5287 (6)	0.8064 (4)	0.170 (4)	
H18A	0.4896	0.5361	0.8043	0.204*	
H18B	0.5772	0.4888	0.8300	0.204*	
C19	0.6121 (14)	0.5788 (7)	0.8425 (6)	0.373 (13)	
H19A	0.6507	0.6052	0.8146	0.559*	
H19B	0.6583	0.5610	0.8763	0.559*	
H19C	0.5603	0.6046	0.8613	0.559*	
C20	0.5707 (5)	0.4523 (3)	0.6077 (2)	0.0720 (17)	
H20A	0.5763	0.4989	0.6055	0.086*	
H20B	0.5941	0.4352	0.5676	0.086*	
C21	0.4580 (5)	0.4354 (3)	0.6107 (3)	0.0768 (17)	
H21A	0.4324	0.4525	0.6502	0.092*	
H21B	0.4499	0.3889	0.6111	0.092*	
C22	0.3964 (5)	0.4635 (3)	0.5524 (3)	0.0824 (19)	
H22A	0.4186	0.4433	0.5134	0.099*	
H22B	0.4110	0.5093	0.5498	0.099*	
C23	0.2801 (5)	0.4539 (3)	0.5552 (3)	0.107 (3)	
H23A	0.2652	0.4088	0.5586	0.161*	
H23B	0.2447	0.4709	0.5165	0.161*	
H23C	0.2569	0.4761	0.5923	0.161*	
C24	0.9918 (4)	0.2829 (3)	0.2790 (3)	0.0779 (18)	
H24A	1.0521	0.2937	0.3076	0.094*	
H24B	1.0118	0.2479	0.2512	0.094*	
C25	0.9651 (5)	0.3400 (3)	0.2371 (3)	0.0866 (19)	
H25A	0.9353	0.3737	0.2626	0.104*	
H25B	0.9143	0.3280	0.2024	0.104*	
C26	1.0645 (6)	0.3650 (4)	0.2085 (4)	0.114 (3)	
H26A	1.1091	0.3841	0.2431	0.137*	
H26B	1.1014	0.3286	0.1916	0.137*	
C27	1.0455 (6)	0.4134 (4)	0.1560 (4)	0.139 (3)	
H27A	0.9949	0.3969	0.1239	0.209*	
H27B	1.1091	0.4220	0.1363	0.209*	
H27C	1.0201	0.4526	0.1739	0.209*	
C28	0.8076 (4)	0.2435 (3)	0.2804 (3)	0.0753 (18)	
H28A	0.7821	0.2831	0.2601	0.090*	
H28B	0.7565	0.2292	0.3095	0.090*	
C29	0.8156 (5)	0.1929 (3)	0.2280 (3)	0.0877 (19)	
H29A	0.8406	0.1528	0.2476	0.105*	
H29B	0.8652	0.2069	0.1979	0.105*	
C30	0.7107 (6)	0.1816 (4)	0.1915 (4)	0.123 (3)	
H30A	0.7160	0.1438	0.1642	0.148*	
H30B	0.6604	0.1719	0.2228	0.148*	
C31	0.6704 (6)	0.2359 (4)	0.1503 (4)	0.137 (4)	
H31A	0.6638	0.2736	0.1767	0.205*	
H31B	0.6037	0.2246	0.1300	0.205*	
H31C	0.7176	0.2446	0.1176	0.205*	
C32	0.8810 (4)	0.3093 (3)	0.3688 (3)	0.0739 (17)	
H32A	0.8554	0.3471	0.3450	0.089*	
H32B	0.8247	0.2932	0.3930	0.089*	
C33	0.9670 (5)	0.3299 (4)	0.4159 (4)	0.110 (3)	
H33A	1.0240	0.3465	0.3927	0.132*	
H33B	0.9922	0.2930	0.4414	0.132*	
C34	0.9291 (6)	0.3817 (5)	0.4606 (4)	0.152 (4)	
H34A	0.9062	0.4191	0.4352	0.183*	
H34B	0.8705	0.3655	0.4826	0.183*	
C35	1.0126 (9)	0.4002 (7)	0.5084 (6)	0.287 (8)	
H35A	0.9871	0.3999	0.5509	0.431*	
H35B	1.0365	0.4427	0.4986	0.431*	
H35C	1.0689	0.3701	0.5069	0.431*	
C36	0.9512 (5)	0.1983 (3)	0.3555 (3)	0.092 (2)	
H36A	0.9692	0.1671	0.3231	0.110*	
H36B	1.0150	0.2104	0.3800	0.110*	
C37	0.8821 (6)	0.1654 (4)	0.4008 (4)	0.110 (3)	
H37A	0.8209	0.1492	0.3762	0.132*	
H37B	0.8596	0.1966	0.4318	0.132*	
C38	0.9358 (7)	0.1103 (5)	0.4369 (5)	0.163 (4)	
H38A	0.8837	0.0831	0.4546	0.196*	
H38B	0.9718	0.0845	0.4064	0.196*	
C39	1.0094 (9)	0.1300 (7)	0.4891 (6)	0.242 (7)	
H39A	1.0576	0.1602	0.4729	0.363*	
H39B	1.0464	0.0930	0.5063	0.363*	
H39C	0.9731	0.1500	0.5227	0.363*	
N2	0.6446 (4)	0.4288 (2)	0.6631 (2)	0.0680 (13)	
N3	0.9076 (4)	0.2582 (2)	0.3203 (2)	0.0719 (14)	

### Atomic displacement parameters (Å^2^)

**Table:**

	*U*^11^	*U*^22^	*U*^33^	*U*^12^	*U*^13^	*U*^23^
Mo1	0.0567 (3)	0.0783 (4)	0.0440 (3)	0.0116 (2)	0.0046 (2)	−0.0007 (2)
Mo2	0.0400 (3)	0.0492 (3)	0.0878 (4)	0.0086 (2)	−0.0024 (2)	0.0086 (2)
Mo3	0.0386 (3)	0.0488 (3)	0.0736 (4)	−0.0025 (2)	−0.0020 (2)	−0.0051 (2)
Mo4	0.0493 (3)	0.0469 (3)	0.0626 (3)	0.0116 (2)	0.0042 (2)	−0.0002 (2)
Mo5	0.0462 (3)	0.0531 (3)	0.0812 (4)	−0.0090 (2)	0.0004 (3)	−0.0035 (2)
Mo6	0.0615 (3)	0.0900 (4)	0.0440 (3)	−0.0026 (3)	−0.0066 (2)	0.0055 (3)
O1	0.089 (3)	0.157 (5)	0.049 (2)	0.027 (3)	0.010 (2)	0.002 (2)
O2	0.059 (2)	0.073 (2)	0.055 (2)	0.0207 (18)	0.0052 (18)	0.0128 (18)
O3	0.061 (2)	0.062 (2)	0.078 (3)	0.0044 (18)	0.022 (2)	0.0173 (19)
O4	0.048 (2)	0.072 (2)	0.060 (2)	0.0024 (18)	−0.0092 (17)	−0.0203 (19)
O5	0.054 (2)	0.064 (2)	0.073 (3)	0.0101 (18)	0.0111 (18)	−0.0099 (19)
O6	0.046 (2)	0.0433 (19)	0.097 (3)	0.0001 (16)	0.0049 (19)	−0.0001 (18)
O7	0.037 (2)	0.061 (2)	0.094 (3)	0.0007 (16)	−0.0030 (19)	0.0051 (19)
O8	0.056 (2)	0.086 (3)	0.066 (3)	0.008 (2)	−0.0114 (19)	0.026 (2)
O9	0.058 (2)	0.059 (2)	0.156 (4)	0.017 (2)	0.008 (2)	0.019 (3)
O10	0.042 (2)	0.056 (2)	0.059 (2)	0.0060 (15)	−0.0009 (16)	−0.0020 (16)
O11	0.048 (2)	0.071 (3)	0.132 (4)	−0.0103 (19)	0.001 (2)	−0.016 (2)
O12	0.061 (2)	0.068 (2)	0.062 (2)	−0.0015 (19)	0.0097 (18)	0.0171 (19)
O13	0.058 (2)	0.046 (2)	0.100 (3)	0.0026 (17)	0.009 (2)	−0.0058 (19)
O14	0.064 (2)	0.073 (2)	0.055 (2)	0.0077 (19)	0.0042 (18)	−0.0123 (18)
O15	0.063 (2)	0.086 (3)	0.071 (3)	−0.011 (2)	−0.015 (2)	−0.018 (2)
O16	0.063 (3)	0.072 (3)	0.152 (4)	−0.023 (2)	0.013 (3)	−0.016 (3)
O17	0.095 (4)	0.177 (5)	0.049 (3)	0.001 (3)	−0.012 (2)	0.014 (3)
O18	0.0372 (18)	0.0427 (18)	0.0454 (19)	0.0011 (13)	−0.0019 (15)	0.0003 (14)
N1	0.059 (3)	0.055 (3)	0.080 (3)	0.015 (2)	0.008 (2)	0.002 (2)
Cl1	0.1314 (18)	0.1374 (18)	0.1341 (18)	0.0128 (14)	0.0456 (14)	−0.0430 (14)
C1	0.056 (3)	0.047 (3)	0.082 (4)	0.008 (3)	0.008 (3)	0.003 (3)
C2	0.073 (4)	0.059 (4)	0.082 (5)	0.001 (3)	0.015 (3)	0.005 (3)
C3	0.080 (5)	0.070 (4)	0.095 (5)	0.016 (4)	0.011 (4)	−0.010 (4)
C4	0.105 (6)	0.066 (5)	0.148 (8)	0.026 (4)	0.024 (6)	−0.008 (5)
C5	0.111 (6)	0.070 (5)	0.125 (7)	0.035 (4)	0.008 (5)	0.039 (4)
C6	0.082 (4)	0.061 (4)	0.082 (5)	0.006 (3)	0.012 (4)	0.009 (3)
C7	0.131 (6)	0.122 (6)	0.088 (6)	0.011 (5)	0.026 (5)	0.017 (5)
C8	0.072 (4)	0.071 (4)	0.074 (4)	−0.022 (3)	−0.019 (3)	0.033 (3)
C9	0.078 (4)	0.064 (4)	0.083 (5)	−0.016 (3)	−0.023 (4)	0.018 (3)
C10	0.071 (5)	0.084 (5)	0.118 (6)	−0.014 (4)	−0.048 (4)	0.031 (4)
C11	0.137 (7)	0.083 (5)	0.153 (8)	0.021 (5)	−0.053 (6)	−0.009 (5)
C12	0.091 (5)	0.074 (4)	0.087 (5)	−0.029 (4)	0.010 (4)	0.021 (4)
C13	0.089 (6)	0.114 (6)	0.140 (7)	−0.041 (5)	−0.006 (5)	0.023 (5)
C14	0.175 (12)	0.217 (14)	0.244 (14)	0.054 (11)	−0.052 (11)	−0.016 (12)
C15	0.266 (19)	0.227 (15)	0.37 (2)	0.081 (13)	0.101 (16)	0.100 (14)
C16	0.107 (5)	0.089 (5)	0.054 (4)	−0.036 (4)	0.001 (4)	0.012 (3)
C17	0.135 (7)	0.128 (7)	0.080 (5)	−0.042 (5)	0.043 (5)	−0.018 (5)
C18	0.177 (10)	0.228 (12)	0.113 (8)	−0.046 (9)	0.057 (7)	−0.037 (8)
C19	0.56 (3)	0.42 (2)	0.166 (13)	−0.28 (2)	0.164 (16)	−0.127 (14)
C20	0.094 (5)	0.068 (4)	0.053 (4)	−0.002 (3)	−0.001 (3)	0.018 (3)
C21	0.085 (5)	0.080 (4)	0.065 (4)	0.008 (4)	−0.002 (3)	0.009 (3)
C22	0.107 (5)	0.072 (4)	0.065 (4)	0.018 (4)	−0.010 (4)	0.003 (3)
C23	0.098 (6)	0.120 (6)	0.100 (6)	0.030 (5)	−0.021 (4)	−0.021 (5)
C24	0.052 (4)	0.091 (5)	0.094 (5)	−0.001 (3)	0.028 (3)	−0.015 (4)
C25	0.065 (4)	0.112 (5)	0.086 (5)	−0.008 (4)	0.025 (4)	−0.017 (4)
C26	0.109 (6)	0.139 (7)	0.100 (6)	−0.001 (5)	0.043 (5)	−0.011 (5)
C27	0.138 (7)	0.188 (9)	0.093 (6)	−0.012 (7)	0.022 (5)	0.018 (6)
C28	0.052 (4)	0.089 (5)	0.086 (5)	−0.002 (3)	0.012 (3)	−0.015 (4)
C29	0.077 (5)	0.100 (5)	0.088 (5)	−0.012 (4)	0.022 (4)	−0.018 (4)
C30	0.112 (7)	0.140 (8)	0.119 (7)	−0.035 (6)	0.030 (5)	−0.034 (6)
C31	0.100 (7)	0.185 (10)	0.123 (8)	−0.006 (6)	−0.005 (6)	0.028 (6)
C32	0.056 (4)	0.082 (4)	0.085 (4)	0.010 (3)	0.016 (3)	−0.014 (4)
C33	0.076 (5)	0.130 (7)	0.123 (6)	−0.018 (5)	0.007 (4)	−0.045 (5)
C34	0.112 (7)	0.202 (10)	0.141 (8)	−0.019 (7)	−0.007 (6)	−0.092 (7)
C35	0.265 (16)	0.34 (2)	0.255 (16)	−0.025 (15)	0.018 (13)	−0.144 (14)
C36	0.065 (4)	0.079 (5)	0.130 (6)	0.014 (4)	0.001 (4)	−0.009 (4)
C37	0.085 (5)	0.102 (6)	0.141 (7)	−0.014 (5)	−0.007 (5)	0.020 (5)
C38	0.107 (7)	0.166 (9)	0.213 (12)	0.009 (7)	−0.013 (7)	0.065 (9)
C39	0.159 (11)	0.38 (2)	0.192 (13)	0.084 (13)	0.053 (9)	0.110 (13)
N2	0.077 (3)	0.068 (3)	0.057 (3)	−0.023 (3)	−0.001 (3)	0.021 (2)
N3	0.053 (3)	0.081 (4)	0.083 (4)	0.007 (3)	0.013 (3)	−0.015 (3)

### Geometric parameters (Å, °)

**Table:**

Mo1—O1	1.674 (4)	C16—C17	1.495 (8)
Mo1—O2	1.911 (3)	C16—H16A	0.9700
Mo1—O5	1.913 (3)	C16—H16B	0.9700
Mo1—O3	1.922 (4)	C17—C18	1.524 (9)
Mo1—O4	1.949 (3)	C17—H17A	0.9700
Mo1—O18	2.321 (3)	C17—H17B	0.9700
Mo2—O9	1.686 (3)	C18—C19	1.399 (8)
Mo2—O7	1.884 (3)	C18—H18A	0.9700
Mo2—O8	1.905 (4)	C18—H18B	0.9700
Mo2—O5	1.921 (4)	C19—H19A	0.9600
Mo2—O6	1.953 (3)	C19—H19B	0.9600
Mo2—O18	2.350 (3)	C19—H19C	0.9600
Mo3—O11	1.682 (3)	C20—C21	1.504 (7)
Mo3—O6	1.901 (3)	C20—N2	1.518 (6)
Mo3—O4	1.916 (3)	C20—H20A	0.9700
Mo3—O12	1.930 (3)	C20—H20B	0.9700
Mo3—O10	1.936 (3)	C21—C22	1.513 (7)
Mo3—O18	2.332 (3)	C21—H21A	0.9700
Mo4—N1	1.732 (4)	C21—H21B	0.9700
Mo4—O2	1.916 (3)	C22—C23	1.520 (8)
Mo4—O10	1.926 (3)	C22—H22A	0.9700
Mo4—O14	1.948 (3)	C22—H22B	0.9700
Mo4—O13	1.980 (3)	C23—H23A	0.9600
Mo4—O18	2.221 (3)	C23—H23B	0.9600
Mo5—O16	1.688 (4)	C23—H23C	0.9600
Mo5—O13	1.863 (3)	C24—C25	1.492 (8)
Mo5—O15	1.916 (4)	C24—N3	1.519 (7)
Mo5—O3	1.932 (4)	C24—H24A	0.9700
Mo5—O7	1.954 (3)	C24—H24B	0.9700
Mo5—O18	2.327 (3)	C25—C26	1.542 (8)
Mo6—O17	1.678 (4)	C25—H25A	0.9700
Mo6—O14	1.882 (3)	C25—H25B	0.9700
Mo6—O12	1.915 (3)	C26—C27	1.486 (9)
Mo6—O8	1.927 (4)	C26—H26A	0.9700
Mo6—O15	1.944 (4)	C26—H26B	0.9700
Mo6—O18	2.332 (3)	C27—H27A	0.9600
N1—C1	1.394 (6)	C27—H27B	0.9600
Cl1—C3	1.744 (6)	C27—H27C	0.9600
C1—C6	1.393 (7)	C28—N3	1.512 (7)
C1—C2	1.404 (7)	C28—C29	1.517 (7)
C2—C3	1.366 (7)	C28—H28A	0.9700
C2—H2	0.9300	C28—H28B	0.9700
C3—C4	1.356 (8)	C29—C30	1.522 (8)
C4—C5	1.371 (9)	C29—H29A	0.9700
C4—H4	0.9300	C29—H29B	0.9700
C5—C6	1.400 (8)	C30—C31	1.483 (9)
C5—H5	0.9300	C30—H30A	0.9700
C6—C7	1.497 (7)	C30—H30B	0.9700
C7—H7A	0.9600	C31—H31A	0.9600
C7—H7B	0.9600	C31—H31B	0.9600
C7—H7C	0.9600	C31—H31C	0.9600
C8—C9	1.493 (8)	C32—C33	1.487 (8)
C8—N2	1.528 (7)	C32—N3	1.516 (6)
C8—H8A	0.9700	C32—H32A	0.9700
C8—H8B	0.9700	C32—H32B	0.9700
C9—C10	1.532 (7)	C33—C34	1.520 (9)
C9—H9A	0.9700	C33—H33A	0.9700
C9—H9B	0.9700	C33—H33B	0.9700
C10—C11	1.481 (10)	C34—C35	1.461 (7)
C10—H10A	0.9700	C34—H34A	0.9700
C10—H10B	0.9700	C34—H34B	0.9700
C11—H11A	0.9600	C35—H35A	0.9600
C11—H11B	0.9600	C35—H35B	0.9600
C11—H11C	0.9600	C35—H35C	0.9600
C12—N2	1.519 (6)	C36—C37	1.504 (9)
C12—C13	1.521 (8)	C36—N3	1.527 (7)
C12—H12A	0.9700	C36—H36A	0.9700
C12—H12B	0.9700	C36—H36B	0.9700
C13—C14	1.707 (13)	C37—C38	1.506 (10)
C13—H13A	0.9700	C37—H37A	0.9700
C13—H13B	0.9700	C37—H37B	0.9700
C14—C15	1.334 (8)	C38—C39	1.445 (13)
C14—H14A	0.9700	C38—H38A	0.9700
C14—H14B	0.9700	C38—H38B	0.9700
C15—H15A	0.9600	C39—H39A	0.9600
C15—H15B	0.9600	C39—H39B	0.9600
C15—H15C	0.9600	C39—H39C	0.9600
C16—N2	1.492 (7)		
			
O1—Mo1—O2	103.89 (18)	C12—C13—H13A	111.8
O1—Mo1—O5	103.37 (18)	C14—C13—H13A	111.8
O2—Mo1—O5	152.67 (14)	C12—C13—H13B	111.8
O1—Mo1—O3	105.59 (19)	C14—C13—H13B	111.8
O2—Mo1—O3	87.17 (15)	H13A—C13—H13B	109.5
O5—Mo1—O3	87.38 (15)	C15—C14—C13	104.1 (12)
O1—Mo1—O4	101.91 (19)	C15—C14—H14A	110.9
O2—Mo1—O4	86.63 (15)	C13—C14—H14A	110.9
O5—Mo1—O4	85.95 (14)	C15—C14—H14B	110.9
O3—Mo1—O4	152.49 (14)	C13—C14—H14B	110.9
O1—Mo1—O18	177.88 (18)	H14A—C14—H14B	109.0
O2—Mo1—O18	75.75 (12)	C14—C15—H15A	109.5
O5—Mo1—O18	76.93 (12)	C14—C15—H15B	109.5
O3—Mo1—O18	76.50 (12)	H15A—C15—H15B	109.5
O4—Mo1—O18	76.00 (12)	C14—C15—H15C	109.5
O9—Mo2—O7	103.98 (16)	H15A—C15—H15C	109.5
O9—Mo2—O8	103.98 (19)	H15B—C15—H15C	109.5
O7—Mo2—O8	88.43 (17)	N2—C16—C17	115.7 (5)
O9—Mo2—O5	103.68 (18)	N2—C16—H16A	108.4
O7—Mo2—O5	87.74 (16)	C17—C16—H16A	108.4
O8—Mo2—O5	152.18 (14)	N2—C16—H16B	108.4
O9—Mo2—O6	103.09 (16)	C17—C16—H16B	108.4
O7—Mo2—O6	152.91 (13)	H16A—C16—H16B	107.4
O8—Mo2—O6	86.00 (15)	C16—C17—C18	107.6 (7)
O5—Mo2—O6	84.98 (14)	C16—C17—H17A	110.2
O9—Mo2—O18	179.04 (15)	C18—C17—H17A	110.2
O7—Mo2—O18	76.95 (12)	C16—C17—H17B	110.2
O8—Mo2—O18	76.22 (12)	C18—C17—H17B	110.2
O5—Mo2—O18	76.08 (12)	H17A—C17—H17B	108.5
O6—Mo2—O18	75.98 (11)	C19—C18—C17	117.4 (9)
O11—Mo3—O6	104.16 (16)	C19—C18—H18A	108.0
O11—Mo3—O4	103.77 (17)	C17—C18—H18A	108.0
O6—Mo3—O4	87.44 (14)	C19—C18—H18B	108.0
O11—Mo3—O12	103.47 (17)	C17—C18—H18B	108.0
O6—Mo3—O12	87.77 (15)	H18A—C18—H18B	107.2
O4—Mo3—O12	152.69 (14)	C18—C19—H19A	109.5
O11—Mo3—O10	102.37 (16)	C18—C19—H19B	109.5
O6—Mo3—O10	153.46 (14)	H19A—C19—H19B	109.5
O4—Mo3—O10	87.06 (15)	C18—C19—H19C	109.5
O12—Mo3—O10	85.33 (14)	H19A—C19—H19C	109.5
O11—Mo3—O18	178.48 (14)	H19B—C19—H19C	109.5
O6—Mo3—O18	77.36 (11)	C21—C20—N2	116.8 (4)
O4—Mo3—O18	76.32 (12)	C21—C20—H20A	108.1
O12—Mo3—O18	76.40 (12)	N2—C20—H20A	108.1
O10—Mo3—O18	76.12 (12)	C21—C20—H20B	108.1
N1—Mo4—O2	103.73 (17)	N2—C20—H20B	108.1
N1—Mo4—O10	104.72 (17)	H20A—C20—H20B	107.3
O2—Mo4—O10	89.98 (15)	C20—C21—C22	109.5 (5)
N1—Mo4—O14	100.37 (18)	C20—C21—H21A	109.8
O2—Mo4—O14	155.58 (14)	C22—C21—H21A	109.8
O10—Mo4—O14	87.68 (14)	C20—C21—H21B	109.8
N1—Mo4—O13	98.72 (17)	C22—C21—H21B	109.8
O2—Mo4—O13	86.63 (15)	H21A—C21—H21B	108.2
O10—Mo4—O13	156.46 (13)	C21—C22—C23	112.6 (5)
O14—Mo4—O13	85.89 (15)	C21—C22—H22A	109.1
N1—Mo4—O18	175.66 (16)	C23—C22—H22A	109.1
O2—Mo4—O18	78.16 (12)	C21—C22—H22B	109.1
O10—Mo4—O18	79.09 (11)	C23—C22—H22B	109.1
O14—Mo4—O18	77.52 (12)	H22A—C22—H22B	107.8
O13—Mo4—O18	77.41 (12)	C22—C23—H23A	109.5
O16—Mo5—O13	104.76 (17)	C22—C23—H23B	109.5
O16—Mo5—O15	102.96 (19)	H23A—C23—H23B	109.5
O13—Mo5—O15	89.82 (16)	C22—C23—H23C	109.5
O16—Mo5—O3	104.03 (19)	H23A—C23—H23C	109.5
O13—Mo5—O3	88.00 (15)	H23B—C23—H23C	109.5
O15—Mo5—O3	152.57 (15)	C25—C24—N3	116.6 (5)
O16—Mo5—O7	101.99 (17)	C25—C24—H24A	108.1
O13—Mo5—O7	153.23 (14)	N3—C24—H24A	108.1
O15—Mo5—O7	85.18 (17)	C25—C24—H24B	108.1
O3—Mo5—O7	84.57 (16)	N3—C24—H24B	108.1
O16—Mo5—O18	178.22 (16)	H24A—C24—H24B	107.3
O13—Mo5—O18	77.01 (12)	C24—C25—C26	108.9 (6)
O15—Mo5—O18	76.71 (13)	C24—C25—H25A	109.9
O3—Mo5—O18	76.17 (12)	C26—C25—H25A	109.9
O7—Mo5—O18	76.25 (12)	C24—C25—H25B	109.9
O17—Mo6—O14	103.84 (19)	C26—C25—H25B	109.9
O17—Mo6—O12	104.67 (19)	H25A—C25—H25B	108.3
O14—Mo6—O12	87.95 (15)	C27—C26—C25	114.1 (7)
O17—Mo6—O8	103.82 (19)	C27—C26—H26A	108.7
O14—Mo6—O8	152.28 (15)	C25—C26—H26A	108.7
O12—Mo6—O8	86.80 (15)	C27—C26—H26B	108.7
O17—Mo6—O15	102.56 (19)	C25—C26—H26B	108.7
O14—Mo6—O15	87.71 (16)	H26A—C26—H26B	107.6
O12—Mo6—O15	152.67 (15)	C26—C27—H27A	109.5
O8—Mo6—O15	84.61 (17)	C26—C27—H27B	109.5
O17—Mo6—O18	178.63 (18)	H27A—C27—H27B	109.5
O14—Mo6—O18	76.03 (12)	C26—C27—H27C	109.5
O12—Mo6—O18	76.69 (12)	H27A—C27—H27C	109.5
O8—Mo6—O18	76.27 (12)	H27B—C27—H27C	109.5
O15—Mo6—O18	76.08 (13)	N3—C28—C29	115.4 (5)
Mo1—O2—Mo4	115.33 (16)	N3—C28—H28A	108.4
Mo1—O3—Mo5	117.23 (16)	C29—C28—H28A	108.4
Mo3—O4—Mo1	117.28 (15)	N3—C28—H28B	108.4
Mo1—O5—Mo2	117.68 (16)	C29—C28—H28B	108.4
Mo3—O6—Mo2	117.32 (16)	H28A—C28—H28B	107.5
Mo2—O7—Mo5	117.61 (17)	C28—C29—C30	111.0 (5)
Mo2—O8—Mo6	118.23 (16)	C28—C29—H29A	109.4
Mo4—O10—Mo3	114.06 (16)	C30—C29—H29A	109.4
Mo6—O12—Mo3	117.35 (16)	C28—C29—H29B	109.4
Mo5—O13—Mo4	114.81 (16)	C30—C29—H29B	109.4
Mo6—O14—Mo4	115.62 (16)	H29A—C29—H29B	108.0
Mo5—O15—Mo6	117.13 (16)	C31—C30—C29	115.6 (7)
Mo4—O18—Mo1	90.73 (10)	C31—C30—H30A	108.4
Mo4—O18—Mo5	90.77 (9)	C29—C30—H30A	108.4
Mo1—O18—Mo5	90.10 (10)	C31—C30—H30B	108.4
Mo4—O18—Mo6	90.77 (10)	C29—C30—H30B	108.4
Mo1—O18—Mo6	178.49 (13)	H30A—C30—H30B	107.4
Mo5—O18—Mo6	89.99 (9)	C30—C31—H31A	109.5
Mo4—O18—Mo3	90.71 (10)	C30—C31—H31B	109.5
Mo1—O18—Mo3	90.34 (9)	H31A—C31—H31B	109.5
Mo5—O18—Mo3	178.45 (13)	C30—C31—H31C	109.5
Mo6—O18—Mo3	89.53 (10)	H31A—C31—H31C	109.5
Mo4—O18—Mo2	179.93 (18)	H31B—C31—H31C	109.5
Mo1—O18—Mo2	89.23 (10)	C33—C32—N3	116.1 (5)
Mo5—O18—Mo2	89.17 (9)	C33—C32—H32A	108.3
Mo6—O18—Mo2	89.27 (9)	N3—C32—H32A	108.3
Mo3—O18—Mo2	89.35 (9)	C33—C32—H32B	108.3
C1—N1—Mo4	169.1 (4)	N3—C32—H32B	108.3
C6—C1—N1	119.5 (5)	H32A—C32—H32B	107.4
C6—C1—C2	121.9 (5)	C32—C33—C34	110.0 (6)
N1—C1—C2	118.6 (5)	C32—C33—H33A	109.7
C3—C2—C1	118.8 (6)	C34—C33—H33A	109.7
C3—C2—H2	120.6	C32—C33—H33B	109.7
C1—C2—H2	120.6	C34—C33—H33B	109.7
C4—C3—C2	121.5 (6)	H33A—C33—H33B	108.2
C4—C3—Cl1	119.0 (5)	C35—C34—C33	110.0 (8)
C2—C3—Cl1	119.5 (6)	C35—C34—H34A	109.7
C3—C4—C5	119.2 (6)	C33—C34—H34A	109.7
C3—C4—H4	120.4	C35—C34—H34B	109.7
C5—C4—H4	120.4	C33—C34—H34B	109.7
C4—C5—C6	123.2 (6)	H34A—C34—H34B	108.2
C4—C5—H5	118.4	C34—C35—H35A	109.5
C6—C5—H5	118.4	C34—C35—H35B	109.5
C1—C6—C5	115.4 (6)	H35A—C35—H35B	109.5
C1—C6—C7	122.0 (6)	C34—C35—H35C	109.5
C5—C6—C7	122.5 (6)	H35A—C35—H35C	109.5
C6—C7—H7A	109.5	H35B—C35—H35C	109.5
C6—C7—H7B	109.5	C37—C36—N3	116.8 (5)
H7A—C7—H7B	109.5	C37—C36—H36A	108.1
C6—C7—H7C	109.5	N3—C36—H36A	108.1
H7A—C7—H7C	109.5	C37—C36—H36B	108.1
H7B—C7—H7C	109.5	N3—C36—H36B	108.1
C9—C8—N2	117.4 (4)	H36A—C36—H36B	107.3
C9—C8—H8A	108.0	C36—C37—C38	112.3 (7)
N2—C8—H8A	108.0	C36—C37—H37A	109.1
C9—C8—H8B	108.0	C38—C37—H37A	109.1
N2—C8—H8B	108.0	C36—C37—H37B	109.1
H8A—C8—H8B	107.2	C38—C37—H37B	109.1
C8—C9—C10	111.9 (6)	H37A—C37—H37B	107.9
C8—C9—H9A	109.2	C39—C38—C37	114.3 (10)
C10—C9—H9A	109.2	C39—C38—H38A	108.7
C8—C9—H9B	109.2	C37—C38—H38A	108.7
C10—C9—H9B	109.2	C39—C38—H38B	108.7
H9A—C9—H9B	107.9	C37—C38—H38B	108.7
C11—C10—C9	114.7 (6)	H38A—C38—H38B	107.6
C11—C10—H10A	108.6	C38—C39—H39A	109.5
C9—C10—H10A	108.6	C38—C39—H39B	109.5
C11—C10—H10B	108.6	H39A—C39—H39B	109.5
C9—C10—H10B	108.6	C38—C39—H39C	109.5
H10A—C10—H10B	107.6	H39A—C39—H39C	109.5
C10—C11—H11A	109.5	H39B—C39—H39C	109.5
C10—C11—H11B	109.5	C16—N2—C20	111.1 (5)
H11A—C11—H11B	109.5	C16—N2—C12	111.9 (4)
C10—C11—H11C	109.5	C20—N2—C12	106.1 (4)
H11A—C11—H11C	109.5	C16—N2—C8	108.5 (4)
H11B—C11—H11C	109.5	C20—N2—C8	110.5 (4)
N2—C12—C13	114.3 (5)	C12—N2—C8	108.7 (5)
N2—C12—H12A	108.7	C28—N3—C32	106.1 (4)
C13—C12—H12A	108.7	C28—N3—C24	112.3 (5)
N2—C12—H12B	108.7	C32—N3—C24	109.9 (4)
C13—C12—H12B	108.7	C28—N3—C36	111.6 (5)
H12A—C12—H12B	107.6	C32—N3—C36	110.3 (5)
C12—C13—C14	99.9 (6)	C24—N3—C36	106.7 (4)

### Hydrogen-bond geometry (Å, °)

**Table:**

*D*—H···*A*	*D*—H	H···*A*	*D*···*A*	*D*—H···*A*
C5—H5···O3^i^	0.93	2.60	3.447 (7)	153.
C8—H8A···O15^ii^	0.97	2.44	3.396 (6)	169.
C9—H9B···O4^iii^	0.97	2.57	3.309 (6)	133.
C16—H16A···O16^ii^	0.97	2.55	3.410 (7)	147.
C7—H7B···N1	0.96	2.42	2.875 (8)	109.
C12—H12A···O9	0.97	2.34	3.248 (7)	155.

Symmetry codes: (i) −*x*+1, −*y*+2, −*z*+1; (ii) −*x*+3/2, *y*−1/2, −*z*+3/2; (iii) −*x*+1, −*y*+1, −*z*+1.
